# A real-world evaluation of radium-223 in combination with abiraterone or enzalutamide for the treatment of metastatic castration-resistant prostate cancer

**DOI:** 10.1371/journal.pone.0253021

**Published:** 2021-06-21

**Authors:** Stephanie I. Kim, Andy H. Szeto, Katherine P. Morgan, Blaine Brower, Mary W. Dunn, Amir H. Khandani, Paul A. Godley, Tracy L. Rose, Ethan M. Basch, Matthew I. Milowsky, Young E. Whang, Daniel J. Crona

**Affiliations:** 1 Division of Pharmacotherapy and Experimental Therapeutics, UNC Eshelman School of Pharmacy, University of North Carolina at Chapel Hill, Chapel Hill, North Carolina, United States of America; 2 Division of Practice Advancement and Experiential Education, UNC Eshelman School of Pharmacy, University of North Carolina, Chapel Hill, North Carolina, United States of America; 3 Department of Pharmacy, UNC Hospitals and Clinics, University of North Carolina at Chapel Hill, Chapel Hill, North Carolina, United States of America; 4 Division of Oncology, Department of Medicine, UNC School of Medicine, University of North Carolina at Chapel Hill, Chapel Hill, North Carolina, United States of America; 5 Division of Molecular Imaging and Therapeutics, Department of Radiology, UNC School of Medicine, University of North Carolina at Chapel Hill, Chapel Hill, North Carolina, United States of America; 6 UNC Lineberger Comprehensive Cancer Center, University of North Carolina at Chapel Hill, Chapel Hill, North Carolina, United States of America; Nine of July University (UNINOVE), BRAZIL

## Abstract

**Introduction:**

Radium-223, abiraterone, and enzalutamide have each been shown to significantly improve survival as monotherapy in patients with metastatic castration-resistant prostate cancer. However, effects of combination radium-223 plus abiraterone or enzalutamide on survival and safety remain unclear.

**Patients and methods:**

This single-center retrospective cohort study used electronic health record data of patients with metastatic castration-resistant prostate cancer and bone metastases who were treated with radium-223 between April 1, 2014 and February 19, 2019. Patients who received radium-223 monotherapy were compared to patients who received a combination of radium-223 plus either abiraterone or enzalutamide. The primary endpoint was overall survival. Secondary endpoints included progression-free survival, time to symptomatic skeletal event, symptomatic skeletal event-free survival, and incidence of drug-related adverse events. Time-to-event analyses were estimated by log rank tests using Kaplan-Meier curves. Hazard ratios and 95% confidence intervals were derived from Cox proportional hazards models. Chi-square tests evaluated difference in serious adverse events between the two arms.

**Results:**

A total of 60 patients met inclusion criteria (n = 41 in the monotherapy arm, n = 19 in the combination arm). Differences in median overall survival were not observed (12.7 vs. 12.8 months; HR 1.15, 95% CI 0.59–2.23; *P* = 0.68), but median progression-free survival was significantly longer in the combination arm (7.6 vs. 4.9 months; HR 1.94, 95% CI 1.11–3.40; *P* = 0.02). Significant differences were not observed in time to first SSE (*P* = 0.97), SSE-free survival (*P* = 0.16), or in the overall incidence of serious adverse events (*P* = 0.45).

**Conclusion:**

Combination radium-223 plus abiraterone or enzalutamide did not improve overall survival, but prolonged progression-free survival without increasing the incidence of serious adverse events in metastatic castration-resistant prostate cancer patients with bone metastases. However, these results are limited by small numbers and patient selection inherent in retrospective analysis.

## Introduction

Prostate cancer is the second leading cause of cancer-related death among men in the United States (U.S.). It is estimated that there will be 191,930 new cases of prostate cancer and 33,330 prostate cancer-related deaths in the U.S in 2020 [1]. Although a significant decline in prostate cancer-related mortality was observed between 1993 and 2015 (approximately 52%), which has been attributed to earlier detection through prostate-specific antigen (PSA) testing and advances in treatment paradigms, prostate cancer-related mortality appears to have plateaued among men younger than age 70 in recent years [1]. While the 5-year survival rates for both local and regional stages of prostate cancer are nearly 100%, the 5-year survival for metastatic prostate cancer is only 29% [2].

Since 2010, seven new therapies (abiraterone, cabazitaxel, enzalutamide, olaparib, radium-223, rucaparib, and sipuleucel-T) have been approved by the U.S. Food and Drug Administration for the treatment of metastatic castration resistant prostate cancer (mCRPC). Among these, abiraterone and enzalutamide, which inhibit the androgen receptor (AR) signaling axis, have been shown to prolong progression-free survival (PFS) and overall survival (OS) in both chemotherapy-naïve patients and patients who have received prior docetaxel chemotherapy [3–6]. Radium-223 dichloride is another treatment option for patients with mCRPC and symptomatic bone metastases without evidence of visceral metastasis, and has been shown to prolong OS [7].

One of the hallmark features of mCRPC is that the disease preferentially metastasizes to bone over visceral organs. Greater than 90% of patients with mCRPC develop osseous metastases, and bone involvement has been consistently associated with skeletal-related events, morbidity, and mortality [8]. Radium-223 is an alpha-emitting radionuclide that mimics calcium and binds hydroxyapatite to form complexes with bone metastases and subsequently induces double-stranded DNA breaks to kill nearby cancer cells [7]. Radium-223 deposits mostly in active bone remodeling areas, accumulating on the bone next to malignant cells and depositing into the bone matrix next to activated osteoblasts. This pattern of deposition suggests potent radiation effects on both tumor cells, osteoclasts, and osteoblasts [9].

Due to the poor prognosis for patients with mCRPC, many medical oncologists have begun to prescribe combination radium-223 with one of the orally administered agents that target the AR signaling axis, despite a lack of data from randomized controlled trials. More recently, the use of combined radium-223 plus abiraterone has been explored, but results from these clinical studies remain conflicting [10–13]. In an international, early access, open-label, single-arm phase 3b trial in radium-223 treated patients, median overall survival was prolonged in patients who received concomitant abiraterone, enzalutamide, or both compared to those who did not receive these agents [13]. A study from the first U.S. expanded access program for radium-223 found that when abiraterone or enzalutamide were added, the combination was well-tolerated with no new identified safety concerns [10]. A retrospective study reported no significant increases in treatment-emergent adverse events (AEs; all grades or grade 3 and higher) [11]. This study also suggested that better Eastern Cooperative Oncology Group Performance Status (ECOG PS), lower reported pain scores at baseline, and normal alkaline phosphatase levels could be predictive biomarkers of survival in patients treated with combination therapy [11]. Similarly, an exploratory analysis of the prospective, non-interventional REASSURE trial showed that patients treated with either concurrent or layered radium-223 plus enzalutamide did not appear to increase the rate of SSEs or fractures [14]. Conversely, the randomized, controlled phase 3 ERA 223 trial found no improvements in SSE-free survival when radium-223 was added to abiraterone compared to abiraterone plus placebo [12]. However, investigators did detect a significantly increased incidence of bone fractures in patients treated with combination of radium-223 and abiraterone [12].

The real-world practice of prescribing radium-223 in combination with other AR-targeting therapies by medical oncologists justifies further investigation into its real-world benefits on survival, but also into whether there could be potential for increased AEs. Therefore, we conducted a single institution retrospective cohort study in mCRPC patients to evaluate the hypothesis that the combination of radium-223 and an inhibitor of the AR signaling axis (i.e., abiraterone or enzalutamide) is associated with prolonged OS or PFS without an increased incidence of serious AEs, when compared to radium-223 alone.

## Methods

### Study design and patient population

This single-center, retrospective study, was approved by the UNC Institutional Review Board (IRB 19–0301). The Carolina Data Warehouse for Health (CDW-H), a central data repository containing clinical, research, and administrative data sourced from the UNC Health Care System, was queried to identify eligible patients treated with at least one cycle of radium-223 between April 1, 2014 and February 19, 2019. Eligible male patients were ≥18 years of age, had histologically confirmed adenocarcinoma of the prostate, had documented osseous metastatic disease with or without radiographic evidence of lymph node involvement (by CT scan, PET imaging, or bone scan), and had undergone prior surgical castration by orchiectomy or medical castration that resulted in testosterone levels <50 ng/dL. Patients had to have confirmed castration-resistant disease, as evidenced rising PSA levels, radiographic or clinical progression while receiving ADT. Patients were excluded if they had evidence of neuroendocrine or small cell histologic features, were diagnosed with a second primary malignancy, or had radiographic evidence of visceral metastatic disease. Patients who had prior exposure to other bone-directed radioisotopes, or were initiated on concomitant cytotoxic chemotherapy or additional investigational agents were also excluded. Eligible patients were stratified into two cohorts: those who received radium-223 monotherapy and those who received either concomitant treatment (both radium-223 and either abiraterone or enzalutamide initiated within 30 days of each other) or layered treatment (either radium-223 or abiraterone/enzalutamide initiated ≥30 days after the first). Hereafter, both concomitant and layered treatments will be referred to as combination treatment.

Data was extracted manually from the institutional electronic medical record (EMR). Relevant clinical data collected included patient demographics (e.g., age, ECOG PS, etc.), past medical history (e.g., osteopenia, osteoporosis), prostate cancer details (e.g., Gleason score), previous and current treatment details (e.g., number of radium-223 cycles, medications and doses, prior use of docetaxel, concomitant use of bisphosphonates or denosumab), and AEs. Relevant laboratory values included basic metabolic panel, complete blood count, prostate cancer-specific markers (e.g., PSA and testosterone), liver function tests (aspartate aminotransferase and alanine aminotransferase, bilirubin, alkaline phosphatase), and information on status of SSEs. Clinical data were collected at baseline prior to the initiation of radium-223, after the final cycle of radium-223, and at progression, as applicable. Follow-up time was measured from time of radium-223 initiation until death, the study ended or the participant was lost, whichever came first.

### Study endpoints

The primary endpoint was OS, which was defined as time from initiation of radium-223 until death of any cause. Patients were censored if they were lost to follow-up or were still alive at the end of the study period. Secondary endpoints included PFS, time to SSE, and AEs that led to dose reductions and/or treatment discontinuations. PFS was defined as time from initiation of radium-223 until evidence of radiographic progression or clinical progression. Patients were censored from time-to-event analyses at the time of last follow-up or if they were switched to a subsequent pharmacotherapeutic agent prior to evidence of progression (e.g., due to intolerable drug-induced toxicity). Similar to the ERA 223 study,^14^ secondary endpoints also included time to PSA response, which was defined as ≥30% reduction in concentrations from baseline.

Time to SSE was defined as time from radium-223 initiation to time of first SSE, defined as use of external beam radiotherapy to relieve skeletal symptoms due to bone metastases, spinal cord compression, or tumor-related orthopedic surgical intervention. AEs were identified through review of physician notes in institutional EMR from the date of radium initiation until 4 weeks following the last radium-223 injection. Severe AEs were defined as those that led to dose reductions, treatment interruptions, or permanent discontinuations.

### Statistical analysis

All statistical testing was two-sided with a significance (alpha) level of 0.05 (*P*<0.05). Descriptive statistics were used to characterize the population of mCRPC patients at UNC. Categorical variables were summarized as counts and percentages, and continuous variables were summarized as medians with the interquartile range (IQR). Means, with 95% confidence intervals were reported when appropriate. Categorical variables were compared across the radium-223 monotherapy arm versus the radium-223 combination arm using a Chi-square of homogeneity test, or Fisher’s exact test, as appropriate. Continuous variables were compared using t-tests when distributions were normal, and Mann-Whitney U tests when sample distributions were not normally distributed. All time-to-event survival analyses (e.g., PFS, OS, time to SSE) were estimated by log rank tests using Kaplan-Meier curves. Hazard ratios and their 95% confidence intervals were derived from Cox proportional hazards models. Logistic regression was used to derive odds ratios and their 95% confidence intervals. All statistical analyses were conducted using SAS JMP version 14.0 (SAS, Cary, NC), and Kaplan Meier curves were generated using GraphPad Prism version 9.0 (GraphPad Software, San Diego, CA, USA).

## Results

### Study population and baseline characteristics

**Fig 1:**
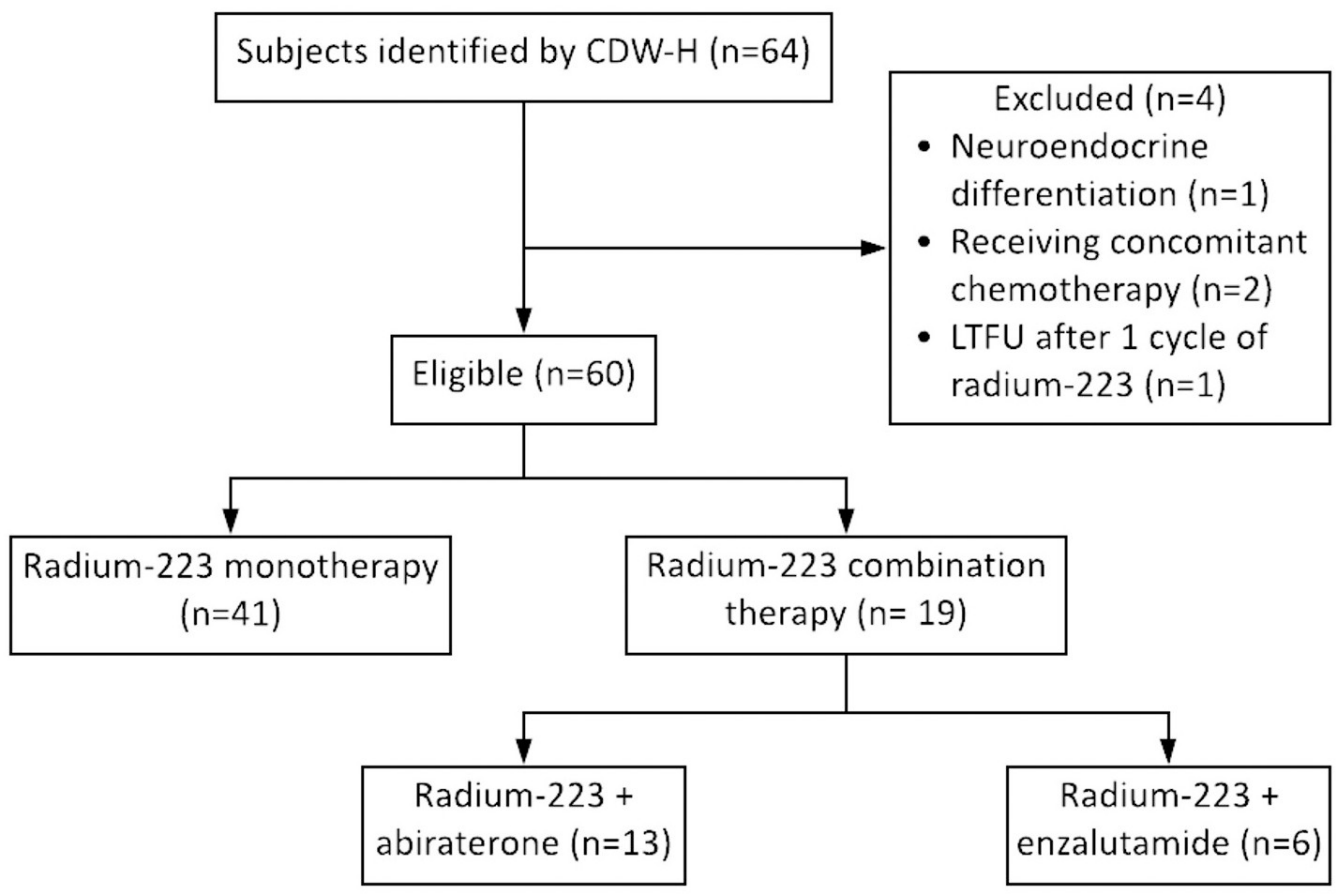
Study schematic. Subjects were identified by CDW-H if they were at least 18 years of age, had an ICD-10 C61 (prostate cancer), and were administered at least one cycle of radium-223 from 4/1/2014 to 2/19/2019. Abbreviations: CDW-H, Carolina Data Warehouse for Health; ICD-10, 10^th^ revision of the International Statistical Classification of Diseases and Related Health Problems; LTFU, lost to follow-up.

A total of 60 patients met the inclusion criteria and were eligible for analysis, with 68% (n = 41) of patients in the radium-223 monotherapy arm and 32% (n = 19) of patients in the radium-223 combination therapy arm (n = 13 treated with radium-223 plus abiraterone and n = 6 treated with radium-223 plus enzalutamide) (Fig 1).

Overall, the baseline patient demographics and clinical characteristics were similar between the two arms, with the exception of median baseline PSA. Baseline PSA was significantly higher in the radium-223 monotherapy arm (101 vs. 29 μg/L; *P* = 0.01) (Table 1). Therefore, only baseline PSA and number of completed radium-223 cycles were evaluated in final multivariate survival models. However, baseline PSA was not independently associated with OS, PFS, SSE-free survival, or time to SSE (all *P*>0.05), and therefore was not included in final survival models. The median age for the entire cohort at the time of radium-223 initiation was 71.5 (IQR 67–80.5) years, and the majority of patients had a Gleason score of 8 or higher (n = 35, 58%). The median number of previous lines of therapy was two. The most commonly used treatment prior to radium-223 was abiraterone (n = 45, 75%), followed by enzalutamide (n = 32, 53%), and docetaxel (n = 19, 32%).

**Table 1:** Patient demographics and clinical characteristics.

Variable	Entire Cohort (n = 60)	Monotherapy (n = 41)	Combination (n = 19)	P-Value
Age, median years (IQR)	71.5 (67–80.5)	71 (67–78)	72 (65–83)	0.91
Race, number (%)				0.58
White	36 (60)	24 (59)	12 (63)	
Black	20 (33)	15 (37)	5 (26)	
Other*	4 (7)	2 (5)	2 (11)	
Gleason score at diagnosis, number (%)				0.40
<8	25 (42)	19 (46)	6 (32)	
≥8	35 (58)	22 (54)	13 (68)	
ECOG performance status, number (%)				0.11
0–1	35 (58)	21 (51)	14 (74)	
≥2	8 (13)	5 (12)	3 (16)	
Missing	17 (28)	15 (37)	2 (11)	
Baseline laboratory measurements, median (IQR)				
PSA (μg/L)**	76 (22–307)	101 (36–466)	29 (12–105)	**0.01**
Alkaline phosphatase (U/L)	157 (92–317)	170 (95–330)	128 (83–317)	0.54
Prior prostatectomy, number (%)	28 (47)	20 (49)	8 (42)	0.78
Prior radiation to prostate, number (%)	28 (47)	21 (51)	7 (37)	0.41
Previous therapy, number (%)				0.81
Abiraterone	45 (75)	32 (78)	13 (68)	
Enzalutamide	32 (53)	21 (51)	11 (58)	
Docetaxel	19 (32)	14 (34)	5 (26)	
Sipuleucel-T	11 (18)	11 (27)	5 (26)	
Ketoconazole	4 (7)	4 (10)	0 (0)	
Apalutamide	1 (2)	1 (2)	0 (0)	
Cabazitaxel	1 (2)	1 (2)	0 (0)	
Previous rounds of therapy, number (%)				0.55
0	3 (5)	2 (5)	1 (5)	
1	23 (38)	16 (39)	7 (37)	
2	15 (25)	8 (20)	7 (37)	
3	11 (18)	8 (20)	3 (16)	
4	8 (13)	7 (17)	1 (5)	
Prior SSE, number (%)	32 (53)	20 (49)	12 (63)	0.41
Medical history of osteoporosis or osteopenia, number (%)	18 (30)	10 (24)	8 (42)	0.23
Concurrent use of denosumab or bisphosphonates, number (%)	31 (52)	20 (49)	11 (58)	0.59

*For the category Race, “Other” includes patients with race not specified in the EMR (n = 3) and Asian (n = 1).

**Baseline PSA was missing for three patients in the combination arm. Percentages were rounded to the nearest integer. P-values <0.05 are bolded. Abbreviations: ECOG, Eastern Cooperative Oncology Group; IQR, interquartile range; PSA, prostate specific antigen; SSE, symptomatic skeletal event.

Among patients in the combination arm, the median duration of overlapping treatment with radium-223 and abiraterone or enzalutamide was 5.8 (IQR 2.8–6.1) months. Of these 19 patients, 15 patients were already prescribed abiraterone or enzalutamide when radium-223 was added onto the treatment regimen, with radium-223 added after at a median of 7.9 (IQR 2.1–12.5) months after initiation of abiraterone or enzalutamide. In the remaining four patients, enzalutamide was initiated in one patient after completion of three cycles of radium-223, while abiraterone was initiated in three patients after completion of one cycle (n = 1) and four cycles (n = 2) of radium-223, respectively. No patients were initiated on combination therapy at the same time, but for three patients radium-223 and abiraterone or enzalutamide were initiated within 30 days of one another. While a significant difference in the number of completed cycles of radium-223 was not observed between the monotherapy versus combination therapy arms (4.4 vs. 5.3; *P* = 0.06) (Table 2), a higher proportion of patients in the combination arm completed all 6 cycles of radium-223 (79% vs 41%; *P* = 0.01).

**Table 2:** Total number of completed radium-223 infusions.

Radium-223 infusions completed, n (%)	Entire Cohort (n = 60)	Monotherapy (n = 41)	Combination (n = 19)	P-Value
6	32 (53)	17 (41)	15 (79)	**0.01**
5	7 (12)	6 (15)	1 (5)	
4	6 (10)	5 (12)	1 (5)	
3	7 (12)	7 (17)	0 (0)	
2	3 (5)	3 (7)	0 (0)	
1	5 (8)	3 (7)	2 (11)	
Mean (SD)		4.43 (0.55)	5.32 (0.56)	0.06

Abbreviation: SD, standard deviation.

### Survival and PSA response

**Fig 2:**
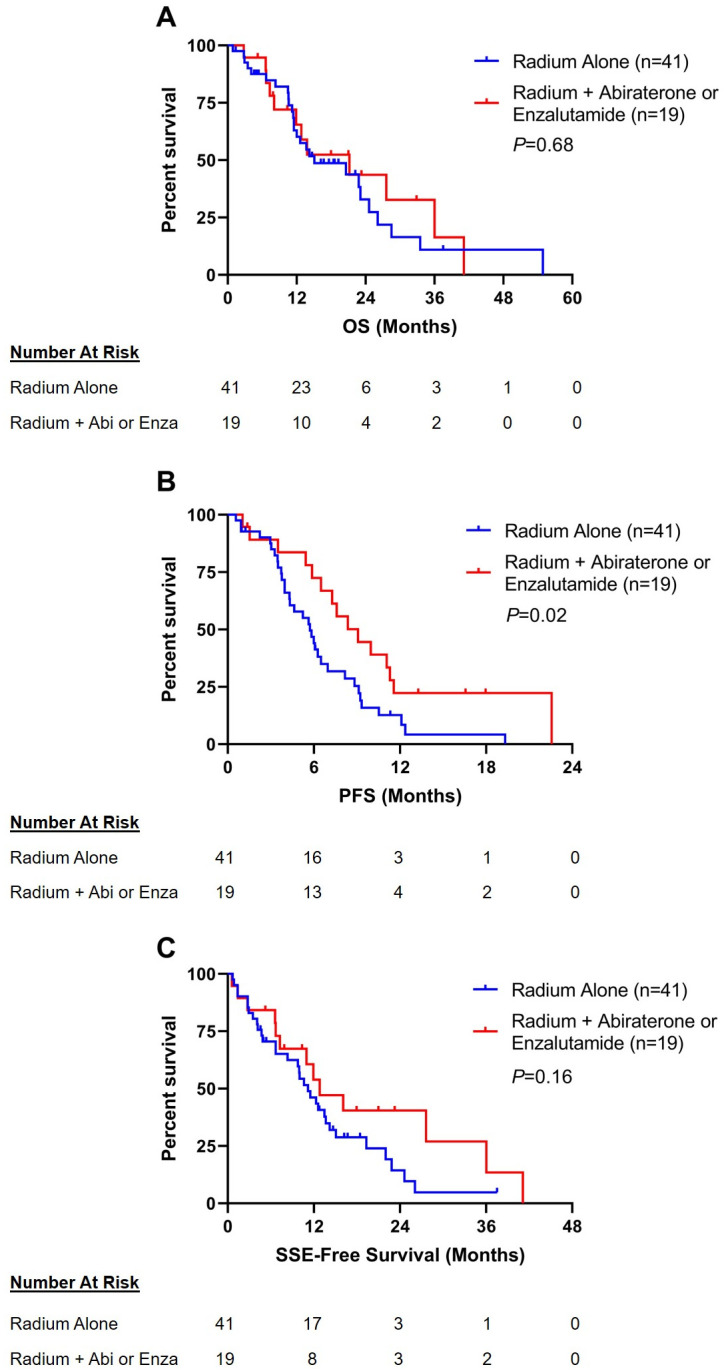
Kaplan-Meier estimates of A) OS, B) PFS, and C) SSE-free survival. Abbreviations: OS, overall survival; PFS, progression-free survival; SSE, symptomatic skeletal event; Abi, abiraterone; Enza, enzalutamide.

The median follow-up time for the entire cohort was 13.3 (IQR 7.3–21.0) months, and was similar between the two arms (13.7 months in the combination arm vs. 12.8 months in the monotherapy arm). Median OS was 12.8 (IQR 7.5–21.1) months and the median PFS was 5.8 (IQR 3.5–9.1) months. Median OS was 12.8 (IQR 7.3–23.3) months in the radium-223 combination arm and 12.7 (IQR 7.5–19.9) months in the radium-223 monotherapy arm, and a significant difference in OS was not detected between the two arms (HR 1.15, 95% CI 0.59–2.23; *P* = 0.68) (Fig 2A). Median PFS was 7.6 (IQR 5.4–11.3) months in the radium-223 combination arm and 4.9 (IQR 3.4–7.6) months in the radium-223 monotherapy arm, and a greater risk of progression was observed in the patients in the radium-223 monotherapy arm than patients in the radium-223 combination arm (HR 1.94, 95% CI 1.11–3.40; *P* = 0.02) (Fig 2B).

**Fig 3:**
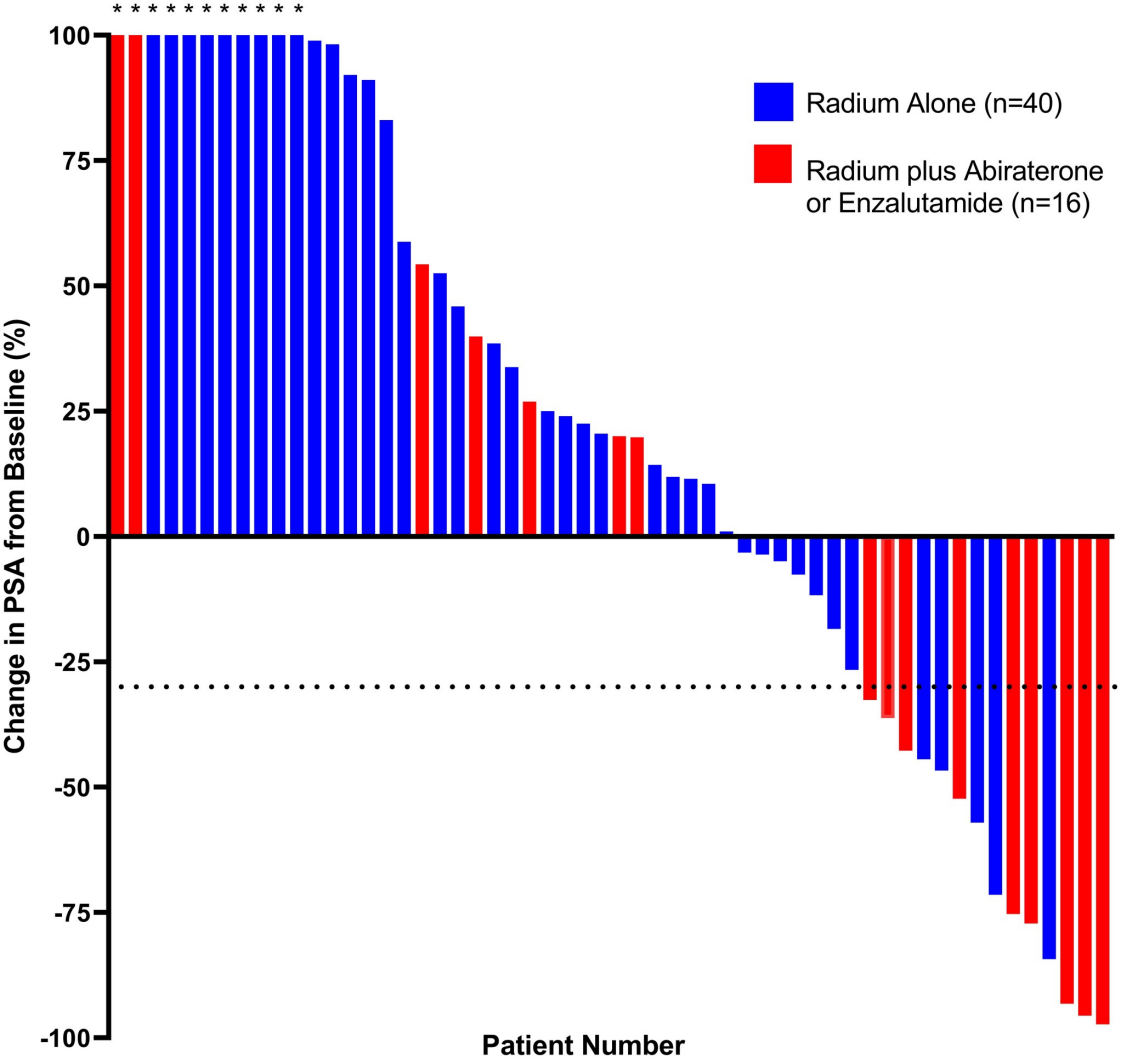
Waterfall plot of best PSA response according to treatment arm. The dotted line depicts the threshold for defining PSA response (≥30% reduction in PSA from baseline). Patients with missing baseline PSA measurements (n = 3), or were lost to follow-up after one cycle of radium-223 (n = 1) were not included in analyses. Asterisks indicate a ≥100% increase in PSA. Abbreviations: PSA, prostate-specific antigen.

A total of 15 patients (25%) in the entire cohort met the criteria for PSA response, with a median time to a ≥30% decrease in PSA of 2.8 (IQR 0.9–4.4) months (Fig 3). PSA response was documented in 47% (n = 9) of the patients in the radium-223 combination arm and 15% (n = 6) of patients in radium-223 monotherapy arm (OR 8.50, 95% CI 2.26–31.7; *P* = 0.002). Median time to PSA response was also shorter among patients in the radium-223 combination arm (1.1 months, IQR 0.9–3.9 months) compared to the radium-223 monotherapy arm (3.6 months, IQR 0.8–5.5 months).

### Symptomatic skeletal events and treatment-emergent adverse events

**Fig 4:**
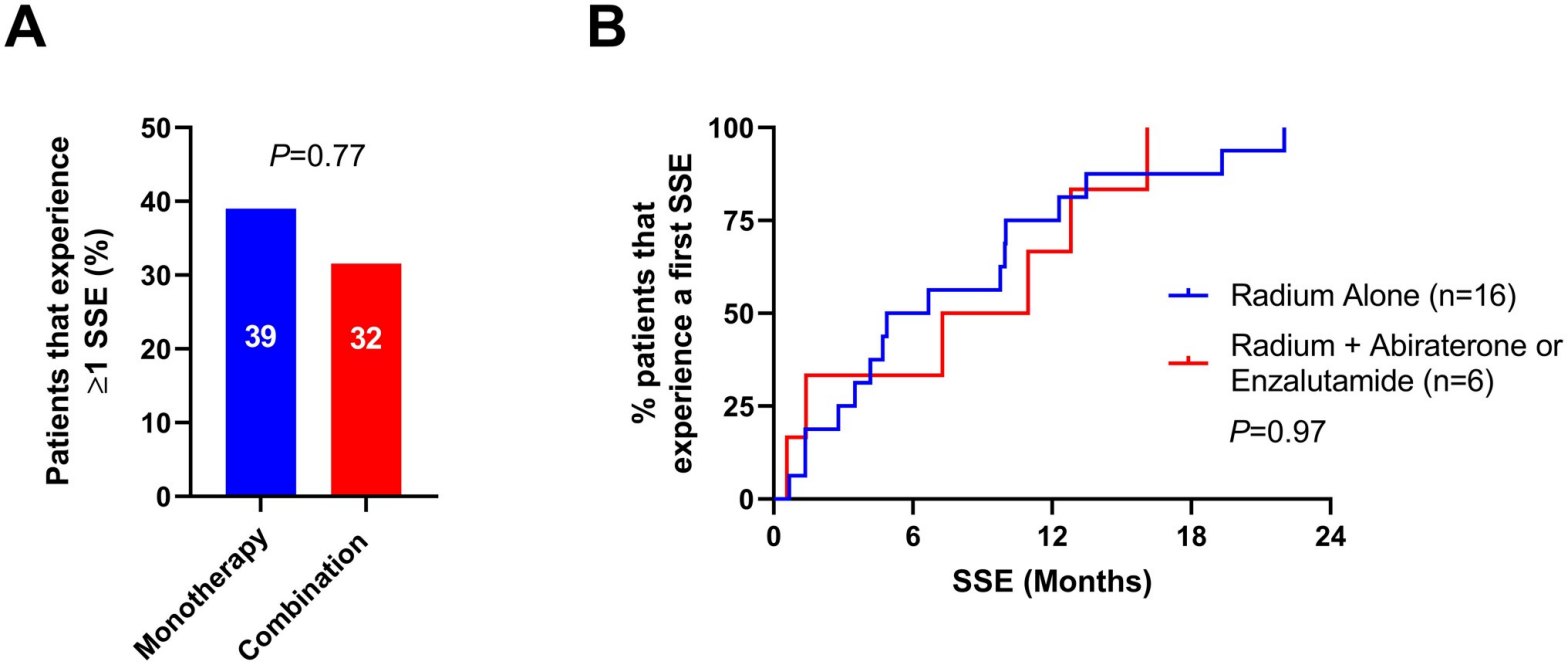
Incidence of and time to first symptomatic skeletal event by treatment arm. Panel **A** shows the percentage of patients who experienced at least one SSE at any point during follow-up in the radium-223 monotherapy arm (n = 41) versus the combination arm (n = 19). Panel **B** depicts the cumulative incidence of patients who experience at least one SSE from time of radium-223 initiation between the monotherapy arm (n = 16) and the combination arm (n = 6). Abbreviations: SSE, symptomatic skeletal event.

The incidence of SSE were similar among the radium-223 monotherapy and combination therapy arms. At least one SSE occurred in 32% (n = 6) of the radium-223 combination arm, and in 39% (n = 16) of patients in radium-223 monotherapy arm (OR 1.39, 95% CI 0.44–4.75; *P* = 0.77) (Fig 4A). First SSEs experienced by patients in this cohort included spinal cord compression (n = 1 [5%] patient in the combination arm vs n = 3 [7%] patients in the monotherapy arm), use of external beam radiation treatment to relieve skeletal symptoms (n = 2 [11%] vs n = 7 [17%]), and pathological fracture (n = 3 [16%] vs n = 6 [15%]). The median time to first SSE was 7.3 (IQR 2.9–12.3) months in the radium-223 combination arm and 4.9 (IQR 3.3–10.6) months in the radium-223 monotherapy arm (HR 1.02, 95% CI 0.40–2.60; *P* = 0.97) (Fig 4B). Last, the median SSE-free survival was 11.0 (IQR 6.6–21.0) months in the radium-223 combination arm and 10.0 (IQR 4.4–14.9) months in the radium-223 monotherapy arm, a significant difference was not detected between the two arms (HR 1.56, 95% CI 0.83–2.92; *P* = 0.16) (Fig 2C).

Table 3 provides a comparison of AEs that occurred in ≥5% of either treatment arm, and AEs that impacted treatment (i.e., led to dose reduction, treatment interruption, or treatment discontinuation). The most common AE among the entire cohort was fatigue or malaise (37%), occurring in 41% of the patients in the radium-223 monotherapy arm and 32% of the combination arm (Table 3). Among patients in the radium-223 monotherapy arm, additional AEs that occurred at a rate ≥20% included bone pain (29%), nausea or vomiting (27%), decreased appetite (27%), back pain (24%), and weight loss (20%). AEs that occurred at a rate ≥20% among patients in the radium-223 combination arm included back pain (37%), nausea or vomiting (37%), diarrhea (32%), bone pain (26%), peripheral edema (26%), anemia (21%), and dyspnea (21%). Only the prevalence of weight loss was significantly different between the two treatment arms, occurring in 20% of patients in the monotherapy arm but 0% in the combination arm (*P* = 0.04). Overall, patients in the radium-223 monotherapy arm experienced a composite of 110 AEs that occurred at a rate ≥5%, compared to a composite of 68 AEs in the radium-223 combination arm (*P* = 0.15). Anemia was the most common serious AE, leading to dose reduction, treatment interruption, or treatment discontinuation in 8% of the total cohort. However, a significant difference in composite AEs that impacted treatment was not observed between the two arms (*P* = 0.45).

**Table 3:** Adverse event profiles between the study arms. AEs that occurred with a frequency ≥5% in at least one of the two study arms and all AEs that impacted treatment are reported. AEs that impacted treatment were defined as those that caused either a dose reduction, treatment interruption, or permanent discontinuation of treatment.

Adverse Event	AEs in ≥5% of patients		AEs that impacted treatment	
	Monotherapy (n = 41)	Combination (n = 19)	Monotherapy (n = 41)	Combination (n = 19)
Fatigue or malaise	17 (41)	6 (32)	2 (5)	1 (5)
Bone pain	12 (29)	5 (26)		
Decreased appetite	11 (27)	2 (11)		
Nausea/Vomiting	11 (27)	7 (37)	1 (2)	0 (0)
Back pain	10 (24)	7 (37)	1 (2)	0 (0)
Diarrhea	8 (20)	6 (32)	0 (0)	1 (5)
Weight loss	8 (20)	0 (0)		
Anemia	7 (17)	4 (21)	4 (10)	1 (5)
Dyspnea	4 (10)	4 (21)		
Peripheral edema	4 (10)	5 (26)		
Constipation	3 (7)	2 (11)		
Pancytopenia	3 (7)	0 (0)	1 (2)	0 (0)
Dizziness	2 (5)	3 (16)		
Hypomagnesemia	2 (5)	0 (0)	1 (2)	0 (0)
Memory impairment	2 (5)	3 (16)		
Asthenia	1 (2)	2 (11)		
Blister lesions	1 (2)	2 (11)		
Hypocalcemia			1 (2)	0 (0)
Insomnia	1 (2)	2 (11)		
Neuropathy	1 (2)	2 (11)		
Upper RTI	1 (2)	2 (11)		
DVT	0 (0)	2 (11)		
Hypertension	0 (0)	2 (11)		
**COMPOSITE**	110	68	11	3

Adverse events are reported as numbers with percentages. Monotherapy refers to radium-223 alone, while combination refers to radium-223 plus abiraterone or enzalutamide. P-values <0.05 are bolded. Abbreviations: AE, adverse event; DVT, deep vein thrombosis; RTI, respiratory tract infection.

## Discussion

The objective of this study was to determine whether the combination of radium-223 plus abiraterone or enzalutamide, when compared to radium-223 alone, would prolong survival without increasing the incidence of severe AEs. The use of combination therapy could be an attractive option for the treatment of mCRPC and bone metastases, but data regarding the use of radium-223 plus abiraterone remain conflicting [7, 11, 12]. In this population of real-world patients, the combination of radium-223 plus either abiraterone or enzalutamide was associated with an increase in PFS, without an increase in SSEs or serious treatment-emergent toxicities.

For the primary endpoint, OS, there was no significant difference detected between the radium-223 monotherapy arm and the combination arm. Consistent with findings from the phase 3 ALSYMPCA trial that led to the approval of radium-223 and demonstrated a median OS of 14.9 months for the radium-223 arm, the median OS for the radium-223 monotherapy arm in the present study was 12.7 months [7]. Moreover, in this present study the observed median PFS for the radium-223 combination arm was significantly longer than the radium-223 monotherapy arm by 2.7 months. However, the clinical significance of this finding is unclear due to a small sample size. Additionally, in the combination arm, most patients (15 out of 19) had radium-223 added on to abiraterone or enzalutamide that they were previously taking. This may introduce selection bias in the combination arm for patients still benefiting from abiraterone or enzalutamide and with less tumor burden, in comparison to the monotherapy arm.

While patients in this study on radium-223 combination therapy had longer PFS than patients on monotherapy, there was no significant difference detected in time to SSE, or in the proportion of at least one SSE, between the radium-223 monotherapy arm and the combination arm. The median time to SSE reported in the radium-223 arm of ALSYMPCA trial (15.6 months) was far greater than the median time to SSE reported in both arms of the present study (9.1 and 5.8 months) [7]. However, the percentage of subjects in the radium-223 arm of the ALSYMPCA trial that experienced an SSE compared to placebo (35% vs. 40%) was similar to the percentages found in the present study (39% monotherapy vs. 32% combination) and ERA 223 (29% abiraterone vs. 30% radium-223 plus abiraterone).

The median OS was 12.8 months for the radium-223 combination arm, which is less than that found in the radium-223 plus abiraterone arm of the ERA 223 trial (30.7 months) [12]. The major difference between the cohort presented in this study and that of the ERA 223 trial was previous therapies at baseline. In the ERA 223 trial, the two treatment arms, abiraterone plus placebo versus abiraterone plus radium-223, were for the first-line treatment of mCRPC, whereas 95% of patients in this study had at least one line of therapy for mCRPC. Less than 2% of the entire cohort of patients in the ERA 223 trial had previous use of docetaxel (all in the hormone sensitive setting), compared to 57% in the ALSYMPCA trial, and 32% in the present study. Optimal timing of radium-223 in the disease course has been proposed as prior to chemotherapy, when performance status is preserved, and before skeletal involvement is widespread, as the bone marrow reserve is still spared [15].

The rate of PSA response in this study among patients in the radium monotherapy arm was consistent with the rate of PSA response among patients from the ALSYMPCA trial (15% vs. 16%, respectively) [7]. However, the rate of documented PSA response was observed in 47% of patients in the radium-223 combination arm. The increased PSA response rate may be due to the additive activity of these agents or potentially synergistic activity.

Overall, treatments with radium-223 alone and in combination with abiraterone or enzalutamide were well-tolerated, where only weight loss was significantly different between the two study arms (Table 3). Interestingly, weight loss was significantly more common in the radium-223 monotherapy arm, which is more likely due to the degree of mCRPC severity and not necessarily a medication effect. The most common AE that led to dose reductions, treatment delays, or permanent treatment discontinuations was anemia, which occurred in 8% of the entire cohort. This finding was consistent with the ALSYMPCA trial, which reported anemia as a serious AE that occurred in 8% and 9% of patients in the radium-223 and placebo groups, respectively. Additionally, anemia of any grade was observed in 18% of this study cohort, which was similar to the rate observed in the ERA 223 trial (13%), but lower than observed in the ALSYMPCA trial (31%). Interestingly, prior docetaxel has been associated with a higher incidence of anemia, across several clinical trials and several retrospective studies, and aligns with the observation that the ALSYMPCA trial had higher rates of both docetaxel pretreated patients and anemia than patients in the present study [7, 12, 16–18].

While these data identified a possible association between radium-223 combination therapy and PFS in a real-world medical oncology practice setting, which provides potential rationale to support future prospective lines of clinical and translational research inquiry, several limitations were present in this study. For example, while abiraterone and enzalutamide are both inhibitors of the AR signaling axis, they have distinct mechanisms of action. With only 19 patients eligible for the combination arm of this real-world retrospective study (13 prescribed abiraterone and 6 prescribed enzalutamide), combining them into one arm could have the potential to confound results. Overall, one of the primary limitations of this study was power to detect an OS benefit in patients treated with radium-223 combination therapy. Future prospective studies with power to detect OS, particularly to evaluate the effects of radium-223 plus enzalutamide, could help discern whether combination therapy is beneficial. This includes the randomized phase III EORTC-1333-GUCG/PEACE III trial (NCT02194842), which seeks to assess radiographic PFS, OS, first SSE, and time and incidence of first skeletal progression-free endpoints with radium-223 plus enzalutamide compared to enzalutamide alone in asymptomatic or mildly symptomatic patients with chemotherapy naive mCRPC [19, 20].

It should be noted that a slightly higher percentage of patients in this study received concomitant bisphosphonates or denosumab (52%), compared to the ALSYMPCA trial (41% of patients) and the ERA 223 trial (41% of patients) [7, 12], which could account for the lower incidence of observed abiraterone- or enzalutamide-induced SSE in this study. Again, future prospective studies in larger populations of real-world mCRPC patients are needed to either confirm these study results or those described by the ERA 223 trial. Results from this study, the ALSYMPCA and ERA 223 trials [7, 12], as well as results from subgroup analyses from the REASSURE study (49% of patients) [14], and a retrospective study of patients in the Flatiron Health database (60% of patients) [21] highlight the potential underutilization of bone protecting agents like denosumab and zoledronic acid despite guideline recommendations supporting their use. Moreover, an interim safety analysis from the PEACE III trial demonstrated the importance of concomitant use of bone protection agents when combination radium-223 is administered. In their interim analyses, the authors showed that the baseline risk of fracture was 13% (5/38 patients), which is consistency with previous reports in mCRPC [22]. When enzalutamide was added to radium-223, the rate increased to 33% (13/39 patients); however, when bone protecting agents were used with combination radium-223 plus enzalutamide, the risk of fracture fell to only 3% (1/33 patients).

In conclusion, there was no difference between radium-223 monotherapy and combination therapy in OS and increased SSEs or treatment-emergent adverse events. However, patients receiving the combination of radium plus abiraterone or enzalutamide had increased PFS, but this finding is limited by small numbers and patient selection inherent in retrospective analysis. This study highlights how real-world considerations for treatment of mCRPC, including the role of bone modifying agents and treatment sequencing, can impact patient treatment outcomes when radium-223 is combined with abiraterone or enzalutamide.
