# Correction: A real-world evaluation of radium-223 in combination with abiraterone or enzalutamide for the treatment of metastatic castration-resistant prostate cancer

**DOI:** 10.1371/journal.pone.0262326

**Published:** 2021-12-31

**Authors:** Stephanie I. Kim, Andy H. Szeto, Katherine P. Morgan, Blaine Brower, Mary W. Dunn, Amir H. Khandani, Paul A. Godley, Tracy L. Rose, Ethan M. Basch, Matthew I. Milowsky, Young E Whang, Daniel J Crona

The following information is missing from the Funding statement: The authors would also like to gratefully acknowledge support provided by the Willis H. Thompson Prostate Cancer Research Fund. The publisher apologizes for the error.

The co-corresponding authors, Young E. Whang and Daniel J. Crona, should be noted as contributing equally to this work.

There are errors in the Author Contributions. The correct contributions are:

**Conceptualization:** Stephanie I. Kim, Andy H. Szeto, Young E. Whang, Daniel J. Crona.

**Data curation:** Stephanie I. Kim, Andy H. Szeto, Young E. Whang, Daniel J. Crona.

**Formal analysis:** Stephanie I. Kim, Andy H. Szeto, Young E. Whang, Daniel J. Crona.

**Investigation:** Stephanie I. Kim, Katherine P. Morgan, Blaine Brower, Mary W. Dunn, Amir H. Khandani, Paul A. Godley, Tracy L. Rose, Ethan M. Basch, Matthew I. Milowsky, Young E. Whang, Daniel J. Crona.

**Project administration:** Young E. Whang, Daniel J. Crona.

**Resources:** Young E. Whang, Daniel J. Crona.

**Supervision:** Young E. Whang, Daniel J. Crona.

**Writing–original draft:** Stephanie I. Kim, Andy H. Szeto, Katherine P. Morgan, Blaine Brower, Mary W. Dunn, Amir H. Khandani, Tracy L. Rose, Ethan M. Basch, Matthew I. Milowsky, Young E. Whang, Daniel J. Crona.

**Writing–review & editing:** Stephanie I. Kim, Andy H. Szeto, Katherine P. Morgan, Blaine Brower, Mary W. Dunn, Amir H. Khandani, Tracy L. Rose, Ethan M. Basch, Matthew I. Milowsky, Young E. Whang, Daniel J. Crona.
